# Factors associated with teachers’ intention to leave their profession: teacher portraits from two European countries

**DOI:** 10.3389/fpsyg.2024.1450424

**Published:** 2024-07-31

**Authors:** Baiba Martinsone, Aušra Rutkienė, Vilma Žydžiūnaite

**Affiliations:** ^1^Department of Psychology, University of Latvia, Riga, Latvia; ^2^Department of Educational Management and Policy, Education Academy, Vytautas Magnus University, Kaunas, Lithuania; ^3^Educational Research Institute, Vytautas Magnus University, Kaunas, Lithuania; ^4^Institute of Pedagogy, University of Silesia, Katowice, Poland

**Keywords:** teachers, well-being, intention to leave profession, stress, burnout

## Abstract

**Introduction:**

Turnover of teachers is an mportant factor that impedes building and maintaining sustainable positive pedagogical practices to facilitate students’ adjustment. The aim of this study was to elicit a portrait of teachers wanting to leave their profession.

**Methods:**

The research sample comprised 784 teachers from two European countries, namely 357 teachers from Latvia and 427 from Lithuania. Teachers were surveyed on their perceived stress, burnout, and intentions to leave their work alongside socio-demographic variables (age and work experience).

**Results:**

It was found that although teachers in both countries reported moderate stress and burnout levels, Lithuanian teachers indicated higher levels of two burnout dimensions, namely exhaustion and inadequacy. However, Latvian teachers indicated significantly higher turnover intentions. The portrait of teachers who intended to leave their profession was different in both countries. An unexpected finding was that Latvian teachers with a higher desire to leave their profession indicated lower stress and burnout rates. They were mostly 45–64 years old and had more than 25 years of work experience. In Lithuania, teachers’ intention to leave their work was reported by older and more experienced teachers experiencing higher stress and burnout.

**Discussion:**

The findings highlight the need to consistently support the professional well-being of educators, both for committed teachers who want to stay in their profession and for those who might experience some detachment from their work at school.

## 1 Introduction

Teachers are an influential school-related factor affecting the quality of education students receive. At the same time, the problem of teacher turnover (intending to leave the teaching profession) is global. Developing and maintaining a positive teaching and learning environment and orientation toward sustainability in education must be taken into account when discussing well-being in educational settings. Working conditions could have a closer relationship with different teachers’ outcomes than their salary or professional training experiences: for instance, the impact of working conditions on job satisfaction and teachers’ intent to leave the profession could be more significant than that of salary. Teacher turnover is a well-known, worrisome, and sensitive issue for schools, the professional community of teachers, students and their families, the education system, and society all over the world (Changying, 2007). Due to a loss of teaching personnel and intellectual resources, schools are under pressure to find the time and resources to rebuild their teaching staff (Barnes et al., 2007; Kelly et al., 2019) and re-train and recruit new teachers (Ryan et al., 2017), which is especially concerning for high-turnover schools. Across Europe, there are increases in teacher shortages and a lack of qualified teachers; moreover, Latvia and Lithuania are among the countries with an aging teaching workforce, particularly in secondary education (European Commission, 2023).

To deal with the growing demands for teachers, there is a proposal to expand teacher education programs and open up alternative routes for other interested professionals to become teachers (Cha, 2008). However, it is equally important to address the issue of the sustainable retention of existing teachers by improving their workplace conditions (Kelchtermans, 2017; Kravale-Pauliņa et al., 2024). Teachers leaving the profession reflects psychological challenges related to low job satisfaction and burnout (Wang et al., 2015). It is important to understand what teachers value in their professional lives since this could predict their intentions to leave their profession (Wang and Klassen, 2023). Based on the perceived usefulness of the teaching career, teachers could be motivated to pursue the teaching profession by two main values: (a) personal utility value, such as job security, autonomy, or prestige, and (b) social utility value, such as making social contributions, helping students, or connecting with colleagues (Klassen et al., 2021). These utility values influence teachers’ perceptions, specifically concerning their fit within the school setting, which further corresponds with their subsequent quitting intentions and decisions.

Research suggests that job satisfaction strongly predicts teachers’ intention to quit the profession (Preechawong et al., 2021). Teachers who feel attached to the profession have a sense of professional belonging, commitment to their work, and intrinsic satisfaction (Aljumah, 2023) and see advancement opportunities in their profession (Judge et al., 2001). Both institutional factors (e.g., dissatisfaction with rewards received, poor relationships within the school community, work overload), insufficient quality of recruitment (Crossman and Harris, 2006), and personal factors (e.g., gender, work experience, and resilience) may lead teachers to leave the profession (Borman and Dowling, 2008; Chan et al., 2008; Ladd, 2011; Hong, 2012; Chesnut and Cullen, 2014).

There is evidence that teaching is a stressful profession (Kyriacou, 2001; Agyapong et al., 2022), and teacher stress has been linked with adverse professional outcomes, including burnout, absenteeism, and attrition (Von der Embse et al., 2016), as well as decrease of teachers’ school connectedness and self-efficacy (Von der Embse and Mankin, 2020). More generally, the ability to cope with stress is related to teachers’ professional well-being (Biggio and Cortese, 2013). The teaching profession demands a lot of effort and patience; however, different processes may affect their work and attitudes toward the profession and may be direct or indirect factors affecting their intention to leave the profession. For example, it is not uncommon for educational policy decisions to change, for teachers to struggle with student misbehavior, or for there to be insufficient support from the school administration in professional, psychological, and leadership terms (Preechawong et al., 2021). Teacher stress can be contagious through social interactions, impairing teacher-student interactions and the school or classroom climate (Guin, 2004; Elovainio et al., 2011), and hence has a negative influence on students’ learning achievements and well-being (Gluschkoff et al., 2017).

Work stress can lead to burnout – a chronic psychological state that develops gradually and includes feelings of exhaustion, cynicism, inadequacy, and reduced personal accomplishment (Hakanen et al., 2006). Teacher burnout can be related to various factors ranging from the social environment (Fiorilli et al., 2017, 2019) and personality factors to the specific nature of the work itself (Vandenberghe and Huberman, 1999). Rajendran et al. (2020) examined teacher burnout and turnover intention in primary and secondary Australian teachers. Job demands (workload and student misbehavior) and the personal demands of the work–family conflict were indirectly related to the intent to leave the teaching profession, mediated through emotional exhaustion. Previous research shows that burnout affects teachers and is associated with the intention to quit the teaching profession and change careers (Federici and Skaalvik, 2012; Pyhältö et al., 2020). Similarly, like chronic stress and low job satisfaction, teacher burnout and prolonged intentions to leave the profession have an indirect negative impact on students’ well-being, engagement, and academic achievements (Bilz et al., 2022).

Evidence from previous research shows a relationship between teachers’ intentions to leave the profession and demographic factors. Male teachers have consistently reported higher intentions to leave the profession than their female colleagues (Wang et al., 2015), whereas female teachers rate the legacy of their work in the profession higher (Guarino et al., 2006). It has been found that younger teachers tend to leave the profession more than older teachers (Borman and Dowling, 2008) and that teachers often leave the profession within the first 5 years of their work (Buchanan et al., 2013), whereas teachers with more professional experience are more determined to stay in the profession (Smith and Ingersoll, 2004; Van Overschelde and Piatt, 2020). However, even teachers with extensive professional experience leave the profession due to dissatisfaction with the school climate, societal disrespect for the teaching profession, psychological exhaustion, and the constant demands placed on teachers (Borman and Dowling, 2008; Clandinin et al., 2015).

Recasting teaching as a collaborative profession, ensuring lifelong professional development and professional autonomy, and engaging teachers in decision-making are crucial factors preventing teachers’ intentions to leave the profession (UNESCO, 2024).

The aim of this study was to explore and compare factors related to teachers’ intention to leave the profession in two European countries, identifying the profile of teachers considering leaving their profession to support evidence-based recommendations for educational policymakers.

## 2 Methodology

### 2.1 Research participants

The sample comprised 784 teachers from two European countries: 427 teachers from Lithuania and 357 from Latvia took part in the research. In both countries, the participants were teachers involved in the Erasmus research project “Teaching to Be.” The participants’ distribution according to gender, education, age, and work experience is presented in Table 1.

**Table 1:** Socio-demographic characteristics of the research participants (*N* = 784).

	Lithuania		Latvia	
	Number	Percentage	Number	Percentage
Gender				
Male	26	6.1	30	8.40
Female	385	90.2	327	91.60
Other	16	3.8		
Education				
BA (non-university)	22	5.2	10	2.80
BA (university)	212	49.6	132	36.97
Master’s	175	41.0	189	52.94
PhD	6	1.4	20	5.60
Other	12	2.7	6	1.68
Age				
21–29 years	31	7.3	31	8.68
30–44 years	139	32.6	86	24.09
45–54 years	149	34.9	124	34.73
55–64 years	90	21.1	89	24.93
65 years or more	11	2.6	15	4.20
Not identified	7	1.6	12	3.36
Work experience				
Up to 1 year	9	2.1	8	2.24
1–5 years	59	13.8	32	8.96
6–10 years	45	10.5	43	12.04
11–15 years	41	9.6	33	9.24
16–20 years	56	13.1	33	9.24
21–25 years	57	13.3	65	18.21
More than 25 years	160	37.5	143	40.06

### 2.2 Procedure

The survey was completed between August and November 2022. Questionnaires were distributed online via www.apklausa.lt and www.pollmill.com. The sample included teachers participating in the Erasmus+ project “Teaching to be.” This paper used data from the pilot study and pre-test to evaluate teachers’ self-reported stress, burnout, and turnover intentions.

### 2.3 Measures

The Perceived Stress Scale (PSS, Cohen et al., 1983; Cohen and Williamson, 1988), identifies how different situations affect perceived stress. The questions on this scale ask about the respondent’s feelings and thoughts during the last month. For each question, they must choose from the following alternatives: 0 – *never,* 1 – *almost never,* 2 – *sometimes*, 3 – *fairly often,* 4 – *very often*. There are two subscales in the PSS: Perceived helplessness (measuring an individual’s feelings of a lack of control over their circumstances or their own emotions or reactions) and Lack of self-efficacy (measuring an individual’s perceived inability to handle problems). Scores on the PSS range from 0 to 40, with higher scores indicating higher perceived stress: scores ranging from 0 to 13 would be considered low stress, 14–26 would be considered moderate stress, and 27–40 would be considered high perceived stress.

The Bergen Burnout Inventory (BBI, Salmela-Aro et al., 2011; Feldt et al., 2013) is comprised of three core dimensions: (1) exhaustion at work, (2) cynicism toward the meaning of work, and (3) sense of inadequacy at work. Exhaustion refers to the draining of emotional energy and feelings of chronic fatigue, cynicism describes having a distant and negative attitude toward one’s job, and reduced professional efficacy refers to the belief that one is no longer effective in fulfilling one’s job responsibilities (Maslach et al., 1996; Maslach and Leiter, 1997). The inventory uses a 6-point rating scale ranging from *strongly disagree* (1) to *strongly agree* (6).

Teachers Turnover Intention Scale (TI, adapted from Hom and Griffeth, 1991; Jaros, 1997) included two items: “I often think about quitting this organization” and “I intend to search for a position with another employer within the next year.” A 5-point rating scale ranging from *strongly agree* (1) to *strongly disagree* (5) was applied. Consequently, lower numbers indicate a higher intention to leave the profession.

All measures were validated in Latvian and Lithuanian languages. They were translated, reviewed, back-translated until equivalence with the original measures was reached (according to Beaton et al., 2000). The reliability of all instruments was checked and Table 2 presents Cronbach’s alphas for all scales. The results from the Lithuanian and Latvian samples demonstrate high internal consistency (>0.7). The numbers are very similar to the Cronbach’s alphas for the original questionnaires. The randomization and the sample split-half method (as additional tool for reliability, which demonstrated correlation >0.8) were used to exclude or control confounding variables.

**Table 2:** Reliability of Perceived Stress Scale, Bergen Burnout Inventory, and Teachers Turnover Intention Scale.

Scale/sub-scale	Original	Lithuanian sample	Latvian sample
STRESS (PSS)			
Perceived helplessness	0.87	0.89	0.85
Lack of self-efficacy	0.74	0.79	0.77
Overall	0.83	0.71	0.81
BURNOUT (BBI)			
Exhaustion	0.70	0.76	0.78
Cynicism	0.82	0.77	0.80
Inadequacy	0.71	0.78	0.74
Overall	0.85	0.88	0.86
TURNOVER INTENTION (TI)	0.82	0.85	0.79

### 2.4 Data analysis

Descriptive statistics were applied for the results’ presentation in counts, percentages, and graphs. The student’s t-test was applied in order to compare variables according to clusters. The chi-square test was applied in order to compare the distribution of groups between countries. The student t-test for independent samples was applied to compare characteristics between Latvia and Lithuania (data normality was confirmed by the Kolmogorov–Smirnov test). Two-step cluster analysis was used for this purpose by identifying homogeneous subgroups presenting similar characteristics (Gelbard et al., 2007; Allen and Goldstein, 2013; Kent et al., 2014). The first step in this approach involves a single processing step in which the raw input data is compressed into a manageable set of sub-clusters. In the second step, a hierarchical clustering approach is used to gradually merge the sub-clusters into larger and larger clusters without the need for another data transfer. The advantage of the two-step analysis is that it tries to determine the optimal number in the first step and then implements it in the second step with k-means and can handle ordinal as well as nominal variables (Kent et al., 2014). The two-step cluster analysis was implemented using SPSS v. 26.0.

## 3 Results

Teachers’ self-reported stress, burnout, and turnover intentions are represented in Table 3. Overall stress was calculated as a sum (range 0–40). The average is 23.26 for Lithuanian teachers and 23.78 for Latvian teachers, so their overall stress level is moderate and very similar in both countries (*t* = 1.390, df = 782, *p* = 0.165). 19.4% (83 respondents) of Lithuanian teachers reported low overall stress, 73.1% (312) moderate stress, and 7.5% (32) high stress. In the sample of Latvian teachers, 16.7% (61) reported low overall stress, 69.4% (248) moderate stress, and 12.8% (46) high stress.

**Table 3:** Comparison of Lithuanian and Latvian teachers’ self-reported stress, burnout, and turnover intentions.

Scale/sub-scale	Lithuanian sample		Latvian sample		Student’s *t*-test
	Mean	Standard deviation	Mean	Standard deviation	*t*
STRESS (PSS)					
Perceived helplessness	13.90	2.77	14.22	3.11	1.523
Lack of self-efficacy	9.36	1.94	9.56	2.23	1.343
Overall	23.26	5.47	23.78	4.90	1.390
BURNOUT (BBI)					
Exhaustion	3.48	1.15	3.31	1.01	2.178*
Cynicism	2.68	1.04	2.79	0.95	1.534
Inadequacy	2.80	1.13	2.62	0.90	2.433*
Overall	2.92	0.95	2.95	0.82	0.468
TURNOVER INTENTION (TI)	3.71	1.17	2.25	1.08	18.02**

*p < 0.05, **p < 0.01.

A different methodology was used for teachers’ burnout calculations, and the results were calculated as averages for sub-scales and a composite score (minimum 1, maximum 6). Generally, Latvian and Lithuanian teachers experience almost the same level of burnout (*t* = 0.468, df = 782, *p* = 0.640). In both countries, the highest scores from all burnout sub-scales are for exhaustion; however, Lithuanian teachers report significantly higher levels of exhaustion and inadequacy compared to Latvian teachers. The lowest rates are for cynicism in Lithuania and for inadequacy in Latvia.

The teachers’ turnover intention was calculated as an average of two items. Lower values mean a higher intention to leave one’s current school and position. The average is 3.71 in Lithuania and 2.25 in Latvia. This means that Latvian teachers reported a higher intention to leave their profession (*t* = 18.02, df = 782, *p* < 0.001).

### 3.1 Cluster analysis

The first cluster analysis included stress (PSS), burnout (BBI), turnover intention (TI), and age. Two-step cluster analysis (with log-likelihood distance measure and Akaike’s information clustering criterion (AIC)) indicated that the average silhouette measure of cohesion and separation was 0.2 (for both samples), which indicates fair cluster quality. Two-step cluster analysis of stress, burnout, and turnover intention increased the average silhouette measure of cohesion and separation to 0.5 for the Latvian and Lithuanian samples.

Analyzing the Latvian model, the most important predictor in clustering was burnout (maximum, indicated as 1), followed by turnover intention (0.85) and perceived stress (0.36). In the Lithuanian sample, the most important predictor was turnover intention (1), followed by burnout (0.69) and stress (0.16).

Auto-clustering indicated that for both samples, the optimal number based on the highest ratio of distance measures is two clusters (the ratio of distance measure was 3.809 for Latvia and 2.849 for Lithuania). This means that clusters of Latvian samples have bigger differences.

All respondents from Latvia were divided into two approximately equal clusters of 176 and 181 teachers. Burnout was the most important factor in both clusters. The least important factor in both clusters was perceived stress level.

**Figure 1:**
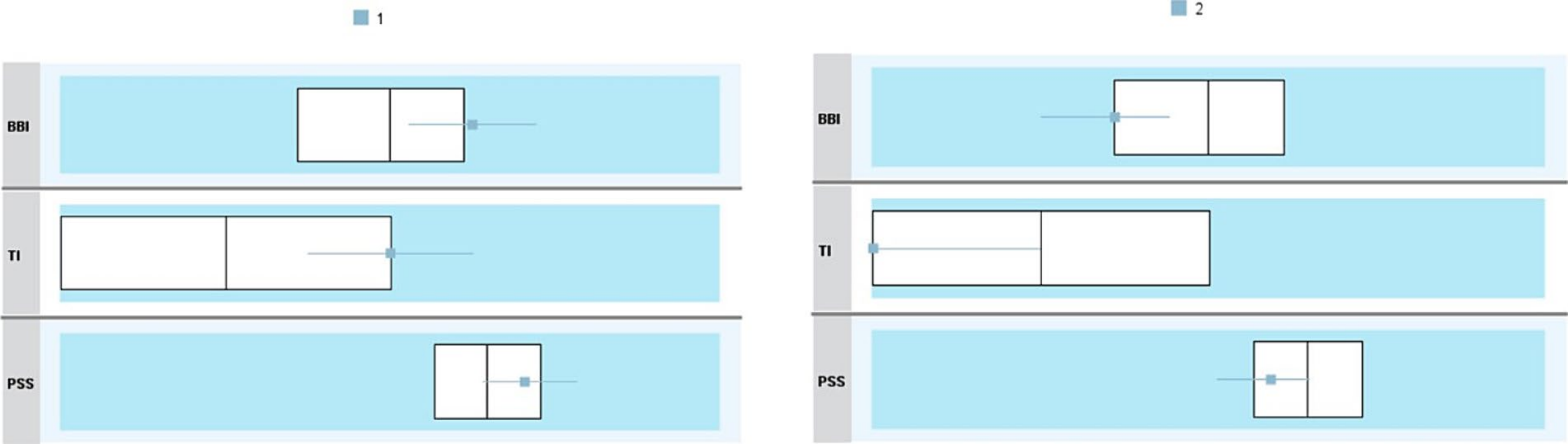
Cluster comparison in Latvian sample.

Figure 1 presents a cluster comparison. Cluster 1 included Latvian teachers with moderate intention to leave their current school and position and comparatively higher stress and burnout levels. In this sample, teachers with higher turnover intention (mean 1.5) have lower burnout and perceived stress levels (Table 4). A student *t*-test (data normality confirmed using the Shapiro–Wilk test) confirmed statistically significant differences between the clusters’ centroids (means difference *p* = 0.000 for all three variables).

**Table 4:** Clusters’ centroids in Latvian sample.

		BBI		PSS		TI	
		Mean	Standard deviation	Mean	Standard deviation	Mean	Standard deviation
Cluster	1	3.556	0.548	26.222	4.303	3.014	0.898
	2	2.354	0.555	21.409	4.233	1.508	0.631
	Combined	2.946	0.816	23.782	4.896	2.251	1.080

**Figure 2:**
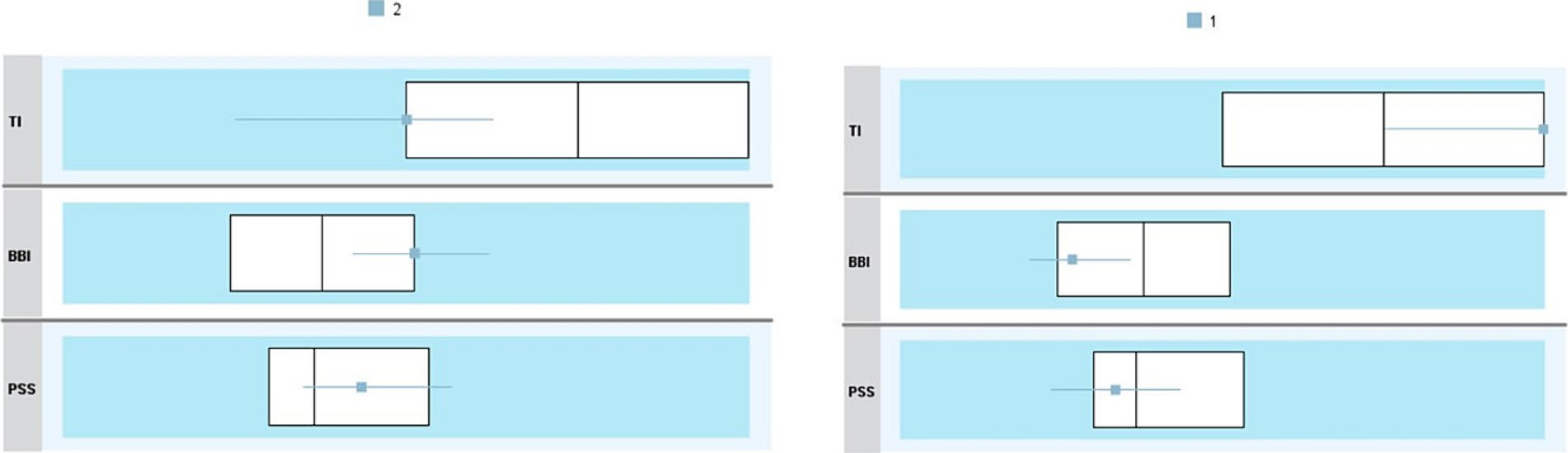
Cluster comparison in Lithuanian sample.

Cluster analysis for the Lithuanian sample demonstrated that the size of both clusters is similar. The first cluster included 228 respondents, and the second included 199 teachers. As the most important variable for clustering Lithuanian teachers was turnover intention, the first cluster had quite a low intention to leave their current position and school, low burnout levels, and moderate perceived stress (Figure 2 and Table 5). The second cluster included teachers with a moderate intention to change workplace, higher burnout, and moderate stress levels.

**Table 5:** Clusters’ centroids in Lithuanian sample.

		BBI		PSS		TI	
		Mean	Standard deviation	Mean	Standard deviation	Mean	Standard deviation
Cluster	1	2.338	0.583	21.474	4.829	4.537	0.576
	2	3.607	0.819	25.312	5.452	2.756	0.920
	Combined	2.929	0.946	23.262	5.469	3.707	1.167

Student’s *t*-test results confirm that the centroids of all three variables in the clusters are significantly different (*p* = 0.000 for all variables).

Analysis of demographics within clusters in the Latvian sample shows that Cluster 1, comprising teachers with higher levels of burnout and stress and a lower intention to leave their work, consists of relatively young but experienced teachers, while Cluster 2 consists of slightly more experienced and older teachers (Table 6).

**Table 6:** Lithuanian and Latvian teachers’ socio-demographic characteristics according to clusters (relative frequencies in %).

	Lithuania		Latvia	
	Cluster 1	Cluster 2	Cluster 1	Cluster 2
Age				
21–29 years	7.5	10.6	6.3	8.3
30–44 years	31.6	33.7	31.8	25.4
45–54 years	35.5	34.2	40.3	32.6
55–64 years	21.5	20.6	19.3	29.8
65 years or more	3.9	1.0	1.1	3.3
Work experience				
Up to 1 year	1.3	3.0	1.1	1.1
1–5 years	12.3	15.6	10.8	7.7
6–10 years	10.5	10.6	14.8	12.7
11–15 years	10.5	8.5	8.0	10.5
16–20 years	11.8	14.6	10.2	11.0
21–25 years	14.0	12.6	20.5	14.9
More than 25 years	39.5	35.2	34.7	42.0

Cluster 1 in the Lithuanian sample was identified as a group with lower burnout, perceived stress level, and turnover intentions, and demographic analysis confirmed that it is a group of slightly older and more experienced teachers. Slightly younger and less experienced teachers in Lithuania experience higher levels of burnout, perceived stress, and turnover intention. No significant differences were observed between clusters according to age (Lithuanian sample chi-square = 2.358, df = 4, *p* = 0.670; Latvian sample chi-square = 5.147, df = 4, *p* = 0.273) or work experience (Lithuanian sample chi-square = 1.891, df = 6, *p* = 0.929, Latvian sample chi-square = 2.628, df = 6, *p* = 0.854).

## 4 Discussion

This study aimed to explore and compare factors related to teachers’ intention to leave the profession in two European countries: Latvia and Lithuania.

It was found that overall stress levels did not differ between the two samples; 73% of Lithuanian and 69% of Latvian teachers reported a moderate level of stress, whereas almost every fifth teacher reported low stress in their professional work. In Lithuania, 7% of teachers reported high perceived stress, but in Latvia, 13% of teachers reported high stress levels. Despite the absence of statistically significant differences between these high stress indicators in both countries, this difference is considerable in the further discussion of the research results. Regarding teachers’ burnout, the fact that teachers report exhaustion more than cynicism is in line with previous research findings (e.g., Martinsone and Žydžiūnaite, 2023). One could speculate that exhaustion is socially more acceptable and teachers are more aware of their physical and emotional exhaustion due to their professional work. Since cynicism is inherently incompatible with the teaching profession, it is likely not easy for teachers to reveal that they distance themselves from their job demands and the needs of students.

There were no differences in teachers’ perceived overall stress and burnout in the two samples; however, Lithuanian teachers reported significantly higher levels of such dimensions of burnout as exhaustion and inadequacy. Compared to Latvian teachers, Lithuanian teachers emphasize their fatigue and perceived inability to fulfill their job responsibilities more. Consequently, the finding that Latvian teachers reported significantly higher intentions to leave their profession than Lithuanian teachers was unexpected. In this research, Latvian teachers emphasized considering leaving their school or even their intention to look for other job opportunities within the next year more often than Lithuanian ones. Consequently, an in-depth analysis was carried out to clarify a portrait of a teacher intending to leave their profession.

The cluster analysis allowed teachers to be grouped according to two qualitatively different groups in each country. In Latvia, burnout was the main variable in which teacher results were the least dispersed. On the other hand, Lithuanian teachers had the smallest individual differences in their intention to leave the profession. About half of the teachers from Lithuania reported high stress and burnout associated with high intentions to leave their jobs. Another group included teachers with low stress and burnout and low turnover intentions. This aligns with the logic of findings from previous research that stress and exhaustion predict teachers’ willingness to leave the profession (Skaalvik and Skaalvik, 2016) and which may also be applied to other professions, for example, health workers (Hämmig, 2018; Christianson et al., 2023), police (Drew et al., 2024) and psychologists (Lidman and Holmberg, 2022).

The two qualitatively different groups of teachers in Latvia were as follows: about half of the teachers reported low stress and burnout, along with high intentions to leave the profession; conversely, the other half pointed out higher rates of stress and exhaustion, along with low intentions to quit their jobs. The former group’s position could be explained by the teachers’ detachment from school, as other studies have also reported (Türktorun et al., 2020; Aulén et al., 2022). In that case, the teacher’s involvement and sense of belonging to the school may be reduced, and the teacher simply ‘survives’ working days. This may result in significant negative long-term consequences and reduce students’ learning achievements and sense of belonging to the school since teachers’ engagement is one of the prerequisites of building a positive school climate (Martinsone et al., 2023) and student adjustment (Gwiazdowska-Stańczak, 2021). The group of teachers who feel stressed and exhausted but do not report turnover intentions should also be respected since tired teachers who stay in their jobs can experience depersonalization and difficulties addressing the needs of both students and themselves, thus negatively impacting long-term outcomes in the social–emotional, behavioral, and academic adjustment of their students.

The following question emerged from these results: what is the socio-demographic profile of each group of teachers? It was found that teacher clusters in Latvia and Lithuania did not differ significantly in terms of teachers’ age and work experience. However, the majority of Latvian teachers reporting higher stress and burnout simultaneously with low turnover intentions were between 30 and 54 years old. They were mostly teachers with notable experience in perhaps their most productive career stages, who, despite their exhaustion, wanted to stay in their profession. This justifies the need to think systematically about promoting teachers’ professional well-being so that they do not accept exhaustion and stress as an integral part of their daily work (Cavioni et al., 2023). Among teachers reporting lower stress and burnout but expressing high turnover intentions were slightly older teachers (up to the age of 64) with more than 25 years of work experience. This portrait of teachers intending to leave their work contradicts other research findings that state younger teachers leave their work more often than older teachers (e.g., Borman and Dowling, 2008). Nevertheless, this finding should be addressed seriously since low levels of stress and burnout combined with a high turnover intention could be explained by the teachers’ detachment from work.

Among Lithuanian teachers, the highest levels of stress, exhaustion, and, consequently, intentions to leave their jobs were reported by slightly younger and less experienced teachers. Conversely, slightly older and more experienced teachers reported lower levels of stress and burnout and low turnover intentions. These results are in line with previous research findings and theoretical considerations that teachers’ burnout and stress are associated with higher turnover rates (Dupriez et al., 2016).

In future research, attention should be paid to work conditions like resources provided, facilities, class sizes, support when disciplining students, school safety, teacher empowerment, and principal leadership (Kimwarey et al., 2014) to keep teachers in schools (Loeb et al., 2005). Also, research is needed to explore ways to support teachers’ competence to sustain their professional well-being.

### 4.1 Conclusion and implications

The current research allowed us to draw several conclusions to provide evidence for building recommendations to reduce teachers’ intentions to leave their profession. First, although Latvian and Lithuanian teachers experience similar levels of stress and burnout, Lithuanian teachers reported higher levels of exhaustion and inadequacy, while Latvian teachers reported significantly higher intentions to quit their jobs. Second, those Lithuanian teachers reporting higher stress and burnout indicated higher turnover intentions. In the Latvian sample, this relationship was the opposite – namely, teachers with lower perceived levels of stress and burnout indicated higher intentions to leave their jobs. Third, there were no differences in age and work experience between Latvian and Lithuanian teachers who reported high turnover intentions.

Consequently, two different portraits of teachers intending to leave their profession were found: the exhausted and stressed teacher wanting to leave their job and the probably detached teacher reporting low levels of stress and burnout but high turnover intentions. It is also crucial to pay attention to the following groups of teachers in all age and work experience categories: (i) exhausted and struggling but want to stay in the profession, (ii) detached and intending to leave their job, and (iii) stressed and with high turnover intentions. Thus, it is of great importance in educational policy and everyday practice to address teachers’ professional well-being to foster teachers’ work engagement, promote a positive school climate, and implement sustainable education.

### 4.2 Strengths and limitations

The large sample of teachers from two European countries and the usage of valid instruments can be considered strengths of this research. Cluster analysis also allowed us to draw qualitatively different profiles of teachers intending to leave their profession. However, the usage of self-report methods may have increased the possibility of socially desirable answers. Moreover, it was not possible to investigate young and inexperienced teachers’ turnover intentions since the vast majority of respondents were teachers with more than 20 years of work experience. Due to the disproportional gender distribution, it was not possible to address gender differences in the teachers’ responses. The research conclusions cannot be applied to the entire teacher sample, because the research participants were teachers from schools involved in the Project “Teaching to Be” not representing all segments of the teacher population in both countries.

Addressing teachers’ intention to leave the profession requires a holistic approach. Factors such as teacher well-being, retention, training, working conditions, and social status need attention. Creating attractive career pathways with equitable access to professional development and autonomy is crucial in sustaining teachers’ motivation. Recognizing the multi-faceted nature of this issue and proposing comprehensive strategies is crucial to finding sustainable solutions.

## Data availability statement

The raw data supporting the conclusions of this article will be made available by the authors, without undue reservation.

## Ethics statement

The studies involving humans were approved by Committee for Educational Research Ethics, Educational Research Institute, Vytautas Magnus University (February 17, 2022, Protocol No. 5) and the Research Board of Vytautas Magnus University (March 1, 2022, Protocol No. 17). The studies were conducted in accordance with the local legislation and institutional requirements. The participants provided their written informed consent to participate in this study.

## Author contributions

BM: Conceptualization, Investigation, Writing – original draft. AR: Investigation, Formal analysis, Writing – original draft. VŽ: Writing – original draft.
